# Are Peripheral Biomarkers Determinants of Eating Styles in Childhood and Adolescence Obesity? A Cross-Sectional Study

**DOI:** 10.3390/nu14020305

**Published:** 2022-01-12

**Authors:** Lorena Desdentado, Jaime Navarrete, María Folgado-Alufre, Ana de Blas, Jéssica Navarro-Siurana, Francisco Ponce, Guadalupe Molinari, Andrea Jimeno-Martínez, Azahara I. Rupérez, Gloria Bueno-Lozano, Aida Cuenca-Royo, Emili Corbella, Zaida Agüera, Rosa M. Baños, Julio Álvarez-Pitti

**Affiliations:** 1 CIBER Fisiopatología Obesidad y Nutrición (CIBEROBN), Instituto de Salud Carlos III, Av. Monforte de Lemos, 3-5. Pabellón 11, Planta 0, 28029 Madrid, Spain; lorena.desdentado@uv.es (L.D.); siscopz80@gmail.com (F.P.); guadalupemolinari@gmail.com (G.M.); andreajimenotic@gmail.com (A.J.-M.); airuperez@unizar.es (A.I.R.); mgbuenol@unizar.es (G.B.-L.); acuenca@imim.es (A.C.-R.); emilic@bellvitgehospital.cat (E.C.); zaguera@bellvitgehospital.cat (Z.A.); banos@uv.es (R.M.B.); 2 Polibienestar Research Institute, University of Valencia, Calle Serpis, 29, 46022 Valencia, Spain; nahijai@uv.es (J.N.); maria.folgado@uv.es (M.F.-A.); jessica.navarro-siurana@uv.es (J.N.-S.); 3 Department of Personality, Evaluation, and Psychological Treatments, University of Valencia, Avda. Blasco Ibañez, 21, 46010 Valencia, Spain; 4 Pediatric Department, Consorcio Hospital General Universitario de Valencia, Avda. Tres Cruces, 2, 46014 Valencia, Spain; adeblaszapata@gmail.com; 5 Growth, Exercise, Nutrition and Development (GENUD) Research Group, Facultad de Ciencias de la Salud, Instituto Agroalimentario de Aragón, Universidad de Zaragoza, Calle Miguel Servet, 177, 50013 Zaragoza, Spain; 6 Paediatric Endocrinology Department, Clinical Hospital Lozano Blesa, Zaragoza, Avda. San Juan Bosco, 50009 Zaragoza, Spain; 7 Integrative Pharmacology and Systems Neurosciences Research Group, Neurosciences Research Program, Hospital del Mar Medical Research Institute (IMIM), 08003 Barcelona, Spain; 8 Cardiovascular Risk Unit, Internal Medicine Department, Bellvitge University Hospital—IDIBELL, Feixa Llarga, s/n, 08907 Barcelona, Spain; 9 Department of Public Health, Mental Health and Perinatal Nursing, Health Sciences Campus Bellvitge, School of Nursing, University of Barcelona, Feixa Llarga, s/n, 08907 Barcelona, Spain

**Keywords:** obesity, children, adolescents, ghrelin, insulin resistance, leptin, adiponectin, leptin/adiponectin ratio, eating behavior, eating styles

## Abstract

Disturbances in eating behaviors have been widely related to obesity. However, little is known about the role of obesity-related biomarkers in shaping habitual patterns of eating behaviors (i.e., eating styles) in childhood. The objective of the present study was to explore the relationships between several biomarkers crucially involved in obesity (ghrelin, insulin resistance, and leptin/adiponectin ratio) and eating styles in children and adolescents with obesity. Seventy participants aged between 8 and 16 (56.2% men) fulfilled the Spanish version of the Dutch Eating Behavior Questionnaire for Children to measure external, emotional, and restrained eating styles. In addition, concentrations of ghrelin, leptin, adiponectin, insulin, and glucose were obtained through a blood test. Hierarchical multiple regression analyses controlling for age and sex were computed for each eating style. Results indicated that individuals with higher ghrelin concentration levels showed lower scores in restrained eating (β = −0.61, *p* < 0.001). The total model explained 32% of the variance of the restrained pattern. No other relationships between obesity-related biomarkers and eating behaviors were found. This study highlights that one of the obesity-risk factors, namely lower plasma ghrelin levels, is substantially involved in a well-known maladaptive eating style, restraint eating, in childhood obesity.

## 1. Introduction

Obesity is a multifactorial disease that is currently considered a public health problem. Specifically, the prevalence of obesity has increased rapidly in childhood and adolescence (5–19 years old), with more than 100 million affected in 2016 [1]. Although recent literature has suggested maintenance, even slight decrease, of childhood obesity prevalence in Spain, it continues to be one of the highest prevalence of obesity and overweight in Europe [2].

These high prevalence figures are partly because there is currently no effective treatment for obesity, which can be generalized to most children and adolescents [3]. Recently, the American Association of Clinical Endocrinologists [4] and the European Association for the Study of Obesity [5] has proposed a new diagnostic term, namely “adiposity-based chronic disease” (ABCD). The phrase “adiposity-based” is justified because the disease is primarily due to abnormalities in the mass, distribution, and/or function of adipose tissue. This fact reinforces the concept of obesity as a chronic, complex, multifactorial, and incurable disease at present. For this reason, it is important to continue exploring the different roots of obesity since this is the only way to develop individualized treatments that ensure a higher success rate.

Eating behaviors stand out as crucial proximal determinants of body weight and the motivation to eat since childhood [6,7]. Eating styles represent dispositional tendencies of food intake and have been closely related to obesity in adults [8] and children and adolescents [9,10]. According to the three main theories about impaired food consumption (i.e., the external, psychosomatic, and restraint theories), three predominant eating styles have been identified [11]. External eating refers to the predominance of external environmental factors, such as smell or sight, as determinants of eating behavior, independently of the internal bodily state (i.e., feeling hungry or satiated) [12,13]. Emotional eating emphasizes the influence of emotional factors so that eating behavior is prompted in response to emotional arousal states, such as fear, anxiety, or anger, again without considering internal physiological signals of hunger and satiety [14]. Restraint eating concerns the attempt to control eating behavior through cognitive control and suppression of internal hunger signals to lose weight or avoid weight gain [13].

Previous research has shown that emotional eating can be considered an “obese” eating pattern, since it has been consistently found that higher emotional eating is related to overweight and long-term weight gain in both adults [8,15,16] and children [17], although the prevalence in childhood (7 to 12 years old) is actually low [11]. Emotional eating seems to emerge in adolescence [18,19] and is more prevalent in females than in males [20].

Restrained eating is also conceptualized as an “obese” eating style [21]. Overall, the distinctive features of restrained eaters are restricting food intake for long periods of time to lose or maintain weight and expressing dissatisfaction towards their body size and/or shape [22]. According to the restraint theory, when the cognitive control of eating is disrupted, restrained eaters tend to show a more disinhibited behavior, increasing their food intake and overeating [23]. Hence, although paradoxical, the involvement of restrained eating in obesity, as well as eating disorders, is not surprising [24].

Unlike emotional and restrained eating, some studies suggest that external eating might not be an “obese eating” pattern (i.e., an eating tendency related to a higher body mass index (BMI)) since levels of external eating seem to be similar between normal-weight and overweight individuals [8,25]. Moreover, it has been argued that external eating could represent an adaptive response that helped survive whenever food is available in periods of food shortage [26]. However, it should be noted that these findings contrast with evidence indicating that overweight and obese individuals show poor interoceptive abilities (i.e., awareness of internal bodily cues accompanying homeostatic states such as satiety) [27], which might predispose them to rely on external cues rather than internal signals of satiety and, therefore, to less adaptive eating behaviors [28]. In this line, Mata and colleagues found that external eating was positively related to the insula activation—which is considered a crucial brain hub for interoceptive processing [29]—in adolescents with excess weight, whereas this relationship was negative among healthy weight adolescents [30]. These results suggest the presence of an altered relationship between insula function and interoceptive/exteroceptive processing in adolescents with excess weight. Moreover, it is known that children with higher reward sensitivity (including sensitivity towards external cues of appetitive stimuli) are more vulnerable to become overweight [31], although this relationship is not straightforward. Specifically, external eating and food responsive behavior mediate the relationship between weight and reward sensitivity in childhood [32].

In the regulation of appetite, not only the central nervous system (CNS), but the adrenal glands, the pancreas, and the gastrointestinal tract are involved, and the adipose tissue also plays a relevant role [33]. Adipose tissue is a metabolically active organ involved in multiple biological processes and communicates through the secretion of peptides and hormones, known as adipokines [34]. As mentioned above, obesity is characterized by a pathological expansion and/or unhealthy distribution of body fat, producing adipose tissue dysfunction. This dysfunction produces a disbalance in the homeostasis of children and adolescents with obesity, favoring a profile characterized by resistance to insulin action (with a secondary elevation of insulin), resistance to leptin action (which favors hyperleptinemia), and low adiponectin and ghrelin values [35]. This profile increases the risk of developing associated cardiometabolic diseases [36] but also could be involved in (maladaptive) eating behaviors in childhood obesity. However, no previous studies have examined this relation.

Ghrelin is a well-known gut hormone involved not only in food intake, but also in energy storage, stimulating adipogenesis [37,38]. Specifically, circulating ghrelin levels increase during intake restriction, leading to increased appetite [39] and then fall quickly after ingestion [39]. Thus, it is postulated that one primary role of ghrelin is to act as a meal initiator. However, to our best knowledge, research on its association with eating styles is scarce in normal-weight [40,41] and obese adults [42] and even absent in obese children and adolescents.

The main metabolic disturbance driven by obesity is insulin resistance (IR) [43]. Insulin levels rise and fall rapidly in response to feeding and starvation. These changing insulin levels orchestrate the metabolic switch between anabolism and catabolism. Glucose is the main regulator of insulin, but also other nutrients, hormones, and the autonomic nervous system, influence its serum levels [44].

As insulin, leptin, which is mostly released by adipose tissue, is well-known to reduce appetite and increase energy expenditure. In obesity, leptin resistance is produced due to the excess adiposity, and, therefore, leptin does not properly reduce food consumption, leading to increased body weight [45]. Circulating leptin levels throughout the day do not undergo large variations, showing a circadian rhythm and oscillatory pattern [46].

Adiponectin is also almost exclusively produced in adipose tissue [47]. The mechanisms of this adipokine in appetite regulation are intricate, and it could be an appetite stimulator or inhibiting factor depending on feeding status, the content of glucose in cerebrospinal fluid, and the degree of fatness [48]. In contrast to other adipokines, the circulating levels of adiponectin are inversely proportional to total fat mass [49], and also with fasting insulin concentration and plasma triglycerides, but positively with the plasma cholesterol contained in HDL [50].

Leptin and adiponectin are regulated in an opposite manner in most cases. Children and adolescents with obesity tend to have higher leptin levels and lower adiponectin levels [51]. This unfavorable leptin/adiponectin ratio has been proposed as a functional biomarker of adipose tissue inflammation and seems to be a good indicator of cardiometabolic risk associated with obesity and metabolic syndrome [52]. However, it remains unknown whether this biomarker acts as an underlying factor of eating styles in obesity.

Thus, eating behavior is modulated by both external and internal signals from the body [53]. External cues include environmental factors such as the hedonic properties of food, as well as social factors such as other people’s behavior [54]. Internal signals usually refer to physiological processes underlying feelings of hunger and satiety, such as the blood concentrations of ghrelin, glucose, and leptin [55]. At this point, it becomes evident that eating behavior and, ultimately, obesity are highly complex. Disentangling the multiple factors involved and the relationships between them can help us better understand obesity and, therefore, develop more effective prevention and treatment methods, especially in the early stages of human development. As pointed out by Verbiest and colleagues [10], interdisciplinary work is needed to do so.

Despite a certain amount of research focused on the regulatory role of these hormonal biomarkers in eating behavior in obese samples, most studies have considered imminent appetite and food intake as outcome variables. However, little is known regarding the contribution of peripheral obesity-related biomarkers to eating styles, that is, to (relatively) well-established patterns of eating behaviors such as emotional, external, and restrained eating.

The aim of this study is to explore the relationship between several peripheral obesity-related biomarkers (i.e., ghrelin, IR, and leptin/adiponectin ratio) and eating styles (i.e., external, emotional, and restrained) in a Spanish sample of obese children and adolescents. Specifically, based on preliminary research on this field, it is hypothesized that poorer obesity-related biomarkers in obese children and adolescents, namely lower ghrelin, higher IR, and higher leptin/adiponectin ratio, will be associated with higher scores on external, emotional, and restrained eating.

## 2. Materials and Methods

### 2.1. Participants

Participants in the current study were recruited from the Obesity and Cardiovascular Risk Unit at General University Hospital Consortium of Valencia (CHGUV) and Pediatric Endocrinology Department at the University Clinical Hospital “Lozano Blesa” (HCULB) in Zaragoza (Spain). Data were collected between November 2019 and September 2021 as a part of the Eat4HealthyLife research project, a multicentered study that explores potential risk factors of obesity and eating disorders (EDs). Eligible criteria considered were the following: aged between 8 and 16 years old, diagnosis of obesity (BMI Z-score > 2 standard deviations according to the scales of their reference group by age and sex), and not having received or not currently receiving obesity treatment in our outpatient clinic. None of the subjects included suffer from any other disease or eating disorder. The sample consisted of 70 children and adolescents with obesity. The mean age of the sample was 12.36 (SD = 2.19), and 56.2% were boys.

### 2.2. Measures

#### 2.2.1. Sociodemographic Data and Anthropometrics

Anthropometric measurements were collected under standardized conditions with patients wearing light clothing and no shoes. Bioelectrical impedance analysis, using a TANITA TBF-410 M over a horizontal and hard surface and to the nearest 0.1 kg, was the selected method to measure body weight and composition. Height was measured to the nearest 0.5 cm using a portable height board (SECA 216). BMI z-score (BMIz) was calculated for each patient. BMIz correlates to growth percentile charts and allows children’s relative weight follow-up from childhood up to adolescence. Participants’ obesity was defined with BMIz scores greater than 2 SD following World Health Organization (WHO) growth charts [56].

#### 2.2.2. Metabolic Assessment

Blood samples were collected from fasting participants scheduled early in the morning. Glucose and insulin measurements and a lipid panel were requested for every patient. The homeostatic model assessment (HOMA) index of IR was measured as the product of insulin (μU/mL) and glucose (mmol/L) divided by 22.5. Hyperinsulinism was defined according to pubertal stage normative data [57].

Adipokine laboratory measurements were obtained from the Cardiovascular Risk Unit Laboratory at Hospital General of Valencia (Valencia, Spain). Ghrelin, leptin, and adiponectin were measured using commercially available BMS 2192, BMS 2039, and BMS 2032/2 eBioscience ELISA kits. The leptin assay detection limit was set at 894 ng/L. Adiponectin assay range was set from 764 μg/L to 16,893 μg/L. The ghrelin assay was set from a mean value of 915 pg/mL.

#### 2.2.3. Eating Styles

The Dutch Eating Behavior Questionnaire for Children (DEBQ-C) [11,58] was used to measure eating styles, which is a 20 item self-report questionnaire rated in a 3-point Likert scale from 1 to 3 (1 = “no”, 2 = “sometimes”, 3 = “yes”) that assess three different eating behavior patterns: external (e.g., “Does walking past a candy store make you feel eating? ”), emotional (e.g., “Does worrying make you feel like eating?”), and restrained (e.g., “Do you intentionally eat food that helps you lose weight?”) eating. Higher scores on each subscale are indicative of greater emotional, external, or restrained eating. Emotional eating and restrained eating subscales are composed of 7 items each, whereas external eating is composed of 6 items. Total scores for each subscale are computed with the mean score of their corresponding items. In this sample, the internal consistency of each scale was acceptable (α = 0.87, α = 0.78, and α = 0.65, respectively). EDs were ruled out according to the diagnostic criteria of the DSM-5 [59].

### 2.3. Procedure

New patients arriving at the Obesity Risk Units were considered potential candidates to participate in this study. At their first appointment at the Unit, participants were weighed and measured to calculate BMIz. Once the patients were diagnosed with obesity, parents were offered the possibility for their children to voluntarily participate in the study. All parents of participants signed the informed consent documents before starting the assessment, in accordance with the Declaration of Helsinki. The study was approved by the Ethics Committee at CHGUV (reference number: 4/2019) and the Research Ethics Committee of the Autonomous Community of Aragon (CEICA) (reference number: PI19/269). Then, patients who agreed to participate were scheduled to come to the hospital in a second session. In this session, participants underwent a blood test to measure biomarkers, and one hour later (after having breakfast), they completed several psychological measures in the context of the Eat4HealthyLife project, including the DEBQ-C, assisted by a trained psychologist. Specifically, the assessment protocol of this project included some neurocognitive measurements, including the Kaufman Brief Intelligence Test Second Edition [60] and the Digit span subtest of the Wechsler Intelligence Scale for Children [61]. However, they are not reported herein because they are not relevant for the objective of this study.

It should be noted that recruitment of participants was stopped during the lockdown period (between March and June 2020), as well as during the third wave of COVID-19 in Spain (between January and March 2021) when the incidence of cases was very high. From the beginning of the pandemic, the study sessions took place with appropriate safety measures, such as mask and ventilation in the room. None of the participants presented symptoms compatible with an infectious process either at the time of the evaluation or in the previous week.

### 2.4. Statistical Analyses

Data analyses were conducted using the SPSS v26 software (IBM, Armonk, NY, USA). First, descriptive statistics (mean, standard deviation) were performed to analyze the sociodemographic data of the sample. Second, Cronbach’s alpha was calculated to establish the internal consistency of the psychometrical measures, with coefficients above 0.70 being considered adequate [62].

Finally, three hierarchical multiple regressions were conducted to determine whether external, emotional, and restrained eating styles (DEBQ-C) were explained by ghrelin, IR, and leptin/adiponectin ratio after controlling for sex and age. Thus, the control variables (sex and age) were entered at Block 1, and the rest of the independent variables were entered at Block 2. Preliminary analyses were performed to check normality, linearity, homoscedasticity, and multicollinearity assumptions, with no serious violations noted.

## 3. Results

### 3.1. Descriptive Statistics

The means, standard deviations, ranges, and percentiles 25, 50, and 75 of the different measures for eating styles and the peripheral obesity-related biomarkers, as well as height and weight, are displayed in Table 1.

### 3.2. Hierarchical Multiple Regressions

#### 3.2.1. External Eating

Regarding the first hierarchical multiple regression model, sex and age explained 5% of the variance of external eating, which did not reach statistical significance: *F* (2, 50) = 1.32, *p* = 0.277, *R*^2^ = 0.050. After entering ghrelin, IR, and leptin/adiponectin ratio at Block 2, the total model did not statistically significantly explain external eating: *F* (5, 47) = 1.25, *p* = 0.303, *R*^2^ = 0.117. Table 2 shows the regression coefficients of the independent variables on external eating.

#### 3.2.2. Emotional Eating

In the second hierarchical multiple regression model, sex and age explained 13% of the variance of emotional eating: *F* (2, 50) = 3.63 *p* = 0.034, *R*^2^ = 0.127. However, after entering ghrelin, IR, and leptin/adiponectin ratio at Block 2, the total model did not statistically explain emotional eating: *F* (5, 47) = 2.36, *p* = 0.054, *R*^2^ = 0.201. Table 3 shows regression coefficients of the independent variables on emotional eating.

#### 3.2.3. Restrained Eating

The third hierarchical multiple regression model showed that sex and age (Block 1) did not significantly explain the variance in restrained eating: *F* (2, 50) = 0.32 *p* = 0.727, *R*^2^ = 0.013. After entry of ghrelin, IR, and leptin/adiponectin ratio at Block 2, the total model statistically significantly explained restrained eating: *F* (5, 47) = 4.37, *p* = 0.002, *R*^2^ = 0.317. Among them, ghrelin was the unique significant predictor of restrained eating (*β* = –0.61; *p* < 0.001), indicating that individuals with higher ghrelin levels showed lower scores on restrained eating, explaining 31% of its variance. Table 4 shows regression coefficients of the independent variables on restrained eating.

## 4. Discussion

The present study was aimed at examining the involvement of different peripheral obesity-related biomarkers (namely, ghrelin, IR, and leptin/adiponectin ratio) to explain different eating styles, i.e., external, emotional, and restrained patterns, in childhood and adolescence obesity.

Our findings show that plasma ghrelin concentrations were negatively related to restrained eating after controlling for sex and age (nonsignificant) effects. In other words, a poor obesity-related biomarker (i.e., lower ghrelin concentrations) was related to restrained eating in childhood and adolescence obesity, as expected. These findings extend previous research that supports the restraint theory [21,23] by suggesting that restrained eating is not only an “obese” eating style, but also a “higher-risk-obesity” eating style. It should be noted that our results contrast with some previous studies conducted in normal-weight adults, indicating that restrained eating is positively correlated [41] or not correlated [40] with ghrelin. According to preliminary research, this discrepancy in the direction of the relationship could be due to the obesity status [63]. Moreover, different measures of restrained eating could reflect distinct conceptualizations of what it means, which might also explain inconsistencies in the literature in this regard. Mainly, scales measuring restrained eating differ in the purpose for which authors developed them [22], i.e., to assess chronic dieters who cyclically restrain their intake or over-eat [64], or individuals who success in reducing their intake [11]. Future studies should be cautious in this concern and simultaneously include individuals with normal and excess weight through different life course stages to disentangle the nature of this relationship. Finally, IR and leptin/adiponectin ratio were not significantly related to restrained eating. In the study conducted by Schur and colleagues [41], leptin and insulin were also measured, but they were not related to cognitive restraint, similar to our results.

External eating was hypothesized to be underlaid by high-risk peripheral biomarkers, specifically lower ghrelin levels, higher IR, and higher inflammation indicators (i.e., leptin/adiponectin ratio). However, our results showed no significant contribution from these biochemical indices to explain external eating once sex and age effects were partialized out. Although there are no previous studies examining the role of ghrelin, IR, leptin, and adiponectin in self-report eating styles in obesity, preliminary evidence exists regarding the influence of ghrelin on the brain activity involved in processing external food cues. In this regard, Malik and colleagues found an increased brain activity response to food-related visual cues after ghrelin administration, which was indeed correlated with self-reported hunger ratings [65]. However, our results suggest that this effect is not extended to the habitual external eating style in childhood obesity.

Similarly, contrary to what was hypothesized, emotional eating was not significantly related to any obesity-related biomarkers in our sample. Sex was the only statistically significant predictor of emotional eating, indicating that women showed higher scores on emotional eating than men, which is consistent with previous findings [20]. Noteworthy in this regard is a previous cohort study that found that higher leptin was cross-sectionally related to emotional overeating at age 7 years, but it was not prospectively associated with emotional overeating three years later after controlling for BMIz. However, this study used a parent-reported instead of a self-report measure of eating styles, that is, parents (or main caregivers) responded to the questionnaire instead of the children on their own (e.g., “My child eats more when annoyed”). Therefore, some biases of their parents could have influenced these results.

To our knowledge, this is the first study to comprehensively consider both physiological risk indicators of obesity and eating styles in a young sample. Theoretically, this study suggests that metabolic dysfunction of adipokines and IR might not yet be involved in eating behavior patterns in obesity in the early stages of development (i.e., childhood and adolescence). This fact promisingly implies that these hormonal disruptions could not have impaired the usual eating responses yet, even if they might be affecting current satiety perceptions, as pointed out by previous research [66]. Given the predictive role of eating styles on prospective weight in adults [15], it can be thought that the clinical condition of obese children and adolescents could still be reversed without consequences in their way of relating to food. In particular, this can be applied to eating styles that involve appropriate processing of interoceptive satiety signals, namely the absence of eating as a maladaptive emotion regulation strategy (emotional eating) and the adaptive levels of sensitivity towards food-related rewards (external eating). However, this is not the case with the cognitive suppression of these internal hunger signals, as evidenced by the negative association between restrained eating and ghrelin levels. In other words, it seems that cognitively ignoring the interoceptive signs of hunger is not a good eating strategy in children and adolescents with obesity, since it is related to a poorer ghrelin profile.

Although interoceptive cues might be ignored at a cognitive level in those with lower ghrelin concentrations, it might be plausible that interoceptive abilities are not yet disturbed in young obese individuals, unlike obese adults [27]. This could explain why emotional and external eating styles were not related to metabolic biomarkers in our sample. Taking all into account, and in light of preliminary research on the role of interoceptive processing as an underlying mechanism of eating styles in adults [67], future studies should examine the interaction between peripheral biomarkers involved in satiety, their perception and processing in the central nervous system (i.e., interoception), and eating styles, in childhood obesity. From a clinical point of view, our findings suggest that dietary treatments mainly focused on restricting the amount of food intake (which inevitably involves ignoring the sensations of appetite) rather than on eating healthy food are contraindicated. In this line, previous research has shown the negative effects of dieting for healthy management of obesity during childhood and adolescence in the long term [68].

The current study presents some limitations that should be noted. First, the sample size was not very large, so the statistical power might be compromised, leading to not finding a statistical effect in this sample that could exist in the population. Second, the age range of participants in this study covered two different developmental stages (childhood and adolescence). Although statistical control has been applied over the potential effect of age (and sex) on the relationships of interest, future studies should replicate this research throughout the different development stages. Third, our results refer only to children and adolescents with moderate and severe obesity (BMIz > 2), so they cannot be generalized to other populations such as children and adolescents with normal weight or overweight (BMIz < 2), nor to adults. Fourth, our findings may be biased by the recruitment period, which was characterized by the COVID-19 pandemic for a portion of our sample. In this regard, a recent longitudinal study showed that external, emotional, and restrained eating styles remained stable during the lockdown in college students [69], supporting the notion of eating styles as trait-like rather than state-like patterns. Although these findings might be different in children and adolescents with obesity, to our best knowledge, there is no evidence to think that the associations between eating styles and biomarkers may vary according to circumstantial factors. Finally, given the cross-sectional nature of the design of the current study, causal relationships cannot be established. Future studies should adopt longitudinal designs to determine the directionality between ghrelin and restrained eating in childhood obesity and the mediating mechanisms by which this relationship occurs.

Despite these limitations, this study contributes substantially to a better understanding of the interactions between two different types of crucial factors (biochemical and psychological) involved in childhood and adolescent obesity, namely circulating hormones and eating styles.

## 5. Conclusions

This is the first study to report the link between the low ghrelin concentrations (which is a well-known biomarker of risk in obesity) and high dispositional tendency to cognitively suppress internal signals of hunger, i.e., restraint eating, which is an eating style considered maladaptive and widely related to obesity across the lifespan. Expanding on previous research, our study indicates that dietary restraint, in addition to being an obesity-related eating style, is also a behavioral pattern of increased risk within the obesity spectrum.

## Figures and Tables

**Table 1:** Descriptive statistics for study measures (*n* = 70).

Measure	M	SD	Range	Percentiles		
				P_25_	P_50_	P_75_
Height (cm)	158.22	11.12	133.90–181.20	151.00	158.40	166.30
Weight (kg)	77.86	18.71	39.80–115.40	64.80	76.70	91.90
External eating	1.99	0.55	1–3	1.50	2.00	2.50
Emotional eating	1.53	0.62	1–3	1.00	1.21	1.86
Restrained eating	1.91	0.45	1–3	1.57	1.86	2.18
Ghrelin (pg/mL)	1497.84	1109.36	90.40–4386.18	690.65	1084.36	2076.84
HOMA index	5.24	5.73	0.89–39.24	2.53	4.14	5.86
Leptin/adiponectin ratio	1.64	2.03	0.04–9.68	0.48	0.94	1.76

Notes. *n* = number of participants; M = mean; SD = standard deviation.

**Table 2:** Regression coefficients of sex, age, ghrelin, IR, and leptin/adiponectin ratio on external eating.

	Model					
Variables	B	SE B	β	*p*	*R* ^2^	∆*R*^2^
Block 1					0.05	0.05
Constant	1.38	0.47		0.005		
Sex	0.22	0.15	0.20	0.164		
Age	0.02	0.04	0.10	0.494		
Block 2					0.12	0.07
Constant	1.60	0.59		0.010		
Sex	0.26	0.16	0.24	0.099		
Age	−0.00	0.04	−0.01	0.967		
Ghrelin	−0.00	0.00	−0.11	0.508		
IR	0.02	0.01	0.19	0.213		
Leptin/adiponectin ratio	0.02	0.04	0.07	0.638		

Notes. B = unstandardized beta values; SE B = standard error of B; β = standardized beta values; *R*^2^ = coefficient of determination; Δ*R*^2^ = coefficient of determination change.

**Table 3:** Regression coefficients of sex, age, ghrelin, IR, and leptin/adiponectin ratio on emotional eating.

	Model					
Variables	B	SE B	β	*p*	*R* ^2^	∆*R*^2^
Block 1					0.13	0.13 *
Constant	0.50	0.51		0.311		
Sex	0.40	0.17	0.32	0.020		
Age	0.04	0.04	0.13	0.318		
Block 2					0.20	0.07
Constant	0.23	0.63		0.713		
Sex	0.42	0.17	0.34	0.015		
Age	0.03	0.04	0.12	0.448		
Ghrelin	0.00	0.00	0.12	0.454		
IR	0.02	0.02	0.19	0.191		
Leptin/adiponectin ratio	0.06	0.04	0.19	0.188		

Notes. B = unstandardized beta values; SE B = standard error of B; β = standardized beta values; *R*^2^ = coefficient of determination; Δ*R*^2^ = coefficient of determination change. * *p* < 0.05.

**Table 4:** Regression coefficients of sex, age, ghrelin, IR, and leptin/adiponectin ratio on restrained eating.

	Model					
Variables	B	SE B	β	*p*	*R* ^2^	∆*R*^2^
Block 1					0.01	0.01
Constant	1.63	0.39		0.000		
Sex	0.08	0.13	0.08	0.558		
Age	0.01	0.03	0.07	0.622		
Block 2					0.32	0.31 **
Constant	2.64	0.42		0.000		
Sex	0.14	0.11	0.16	0.198		
Age	−0.05	0.03	−0.23	0.102		
Ghrelin	0.00	0.00	−0.61	<0.001		
IR	0.01	0.01	0.09	0.506		
Leptin/adiponectin ratio	−0.01	0.03	−0.04	0.758		

Notes. B = unstandardized beta values; SE B = standard error of B; β = standardized beta values; *R*^2^ = coefficient of determination; Δ*R*^2^ = coefficient of determination change. ** *p* < 0.01.

## Data Availability

The dataset generated and analyzed is available from the corresponding author on reasonable request.
